# Technical tips to perform safe and effective Ultrasound Guided steroid joint injections in children

**DOI:** 10.1186/1546-0096-13-2

**Published:** 2015-01-07

**Authors:** Dimitri A Parra

**Affiliations:** Pediatric Interventional Radiologists, Image Guided Therapy, Diagnostic Imaging, The Hospital for Sick Children, Toronto, ON Canada; Department of Medical Imaging, University of Toronto, Toronto, Canada

**Keywords:** Ultrasonography, Steroid injections, Triamcinolone hexacetonide, JIA

## Abstract

**Background:**

The aim of this article is to describe the technique used to perform ultrasound guided steroid joint injections in children in a group of joints that can be injected using ultrasound as the only image guidance modality.

**Findings:**

The technique is described and didactic figures are provided to illustrate key technical concepts.

**Conclusion:**

It is very important to be familiar with the sonographic appearance of the pediatric joints and the developing bone when performing ultrasound-guided joint injections in children.

## Introduction

Juvenile idiopathic arthritis (JIA) is the most common chronic rheumatic disease in childhood with an incidence that ranges from 1 to 22 per 100,000 [1, 2]. Different treatment options are available for this condition (including anti-inflammatory medications and anti-TNF agents), which are selected according to the presentation and course of the disease. Intra-articular steroid joint injections are considered effective and safe for local treatment of arthritis, particularly in patients with oligoarthritis [3, 4]. Even though some joints can be injected using clinical landmarks, Ultrasound (US) provides direct visualization of the joint space, real time needle guidance, can guide therapy according to signs of inflammation and permits the visualization of the adequate distribution of the medication within the joint space. It is being currently accepted in our center, as well as in the literature [5], as one of the preferred method to deliver this therapy in different joints. US can be used alone or in conjunction with fluoroscopy or cross sectional imaging according to the joint of interest.

Due to the widespread use of US in the routine clinical practice of different specialties, including rheumatology [5], the aim of this article is to show the interventional radiology technique employed in our center to perform US guided steroid joint injections in children, in a group of joints in which this may be the only imaging guidance modality needed.

## Findings

### Injection technique

The injections are performed in the Interventional Radiology department in collaboration with the Rheumatology division. They manage the patients with JIA and decide who the ones that require image guided joint injections are. This group is referred to interventional radiology, with the specific joints and doses of steroid to be injected. We inject more than 250 joints a year.

The day of the procedure the patient is re-examined by a rheumatologist and it is decided if the joints requested are still active and if there are new sites that may require treatment. The need for sedation versus general anesthesia is assessed according to the age of the patient and the number of joints to be injected. All injections are performed under a strict sterile technique. Informed consent is obtained before the procedure and the sites to be injected are marked on the skin. In the majority of cases the pediatric joints do not show effusion in US, therefore they are injected according to the clinical symptoms.

In our institution we inject a wide variety of joints using a variety of imaging modalities. We utilize US alone in the joints in which the intra-articular position of the needle as well as the injection of the medication can be clearly seen by this method. This article focuses in these group of joint which include: ankle, knee, hip, radio-carpal and inter-phalangeal joints. A combination of US and fluoroscopy (with injection of iodinated contrast) is used in the shoulder, elbow, carpal, subtalar and mid-foot joints. US is combined with cross sectional imaging (Computed Tomography (CT) or C-arm CT) in the injection of the temporomandibular joints and sacroiliac joints.

The steroid that we use is long-acting triamcinolone hexacetonide (Aristospan^R^, Sandoz Canada Inc, 20 mg/ml) and is injected preferably with 25 gauge 1 1/2 inches needles (Precision Glide Needle, Becton Dickinson & Co, Franklin Lakes, NJ). The needle can be flushed with local anesthetic prior to the injection to avoid air injection that will decrease tip visualization. Luer lock or slip syringes can be used. Slip syringes may help trainees to understand that no pressure is necessary when injecting the steroid into the joint space. If the needle touches the bone or cartilage or if there is resistance to the injection of the steroid, the needle is slightly withdrawn and gentle injection of the medication under direct US visualization is performed to make sure that there is no extravasation of the steroid into the adjacent soft tissues. We routinely inject a small amount of local anesthetic (Lidocaine 1%) after the steroid to provide rapid release of pain/discomfort and to push the steroid into the joint space. Over distention of the joint should be avoided to prevent or diminish post procedural pain.

Potential complications of the procedure include pain, bleeding, infection, skin atrophy, blood/nerve damage, and joint calcifications. The physician performing the procedure should be aware of the main vascular and neural structures located close to the joints to be injected.

### Sonographic appearance of the developing bone/joint

US is very useful in the assessment of the immature bone, especially in the cartilaginous epiphysis. This area appears as a homogeneous hypoechoic or anechoic structure, limited by thin, sharp and slightly hyperechoic margins [6, 7]. The ossification center is central and predominantly echogenic [6], with a size that varies according to the age of the patient. Normal articular cartilage is homogeneous with a sharp interface with the adjacent hyperechoic bone. The joint cavities and articular capsules are barely seen or invisible in ultrasound in normal conditions. Ligaments are seen as echogenic fibrilar structures. Peripheral nerves are seen as hypoechoic fascicles surrounded by a thin echogenic layer. Tendons appear as echogenic fibrillar structures with multiple parallel lines in the longitudinal plane and multiple “dotlike” echoes in the transverse plane [7].

**Figure 1:**
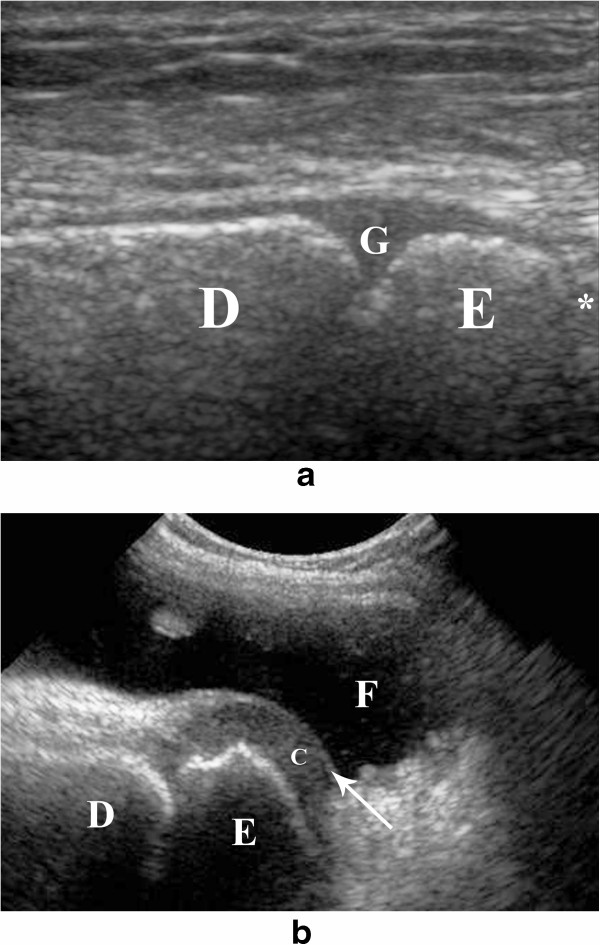
**Sonographic appearance of the developing bone. a**. Longitudinal sonographic view of the distal tibia showing the distal growth plate **(G)** as a hypo-echoic area, in between the diaphysis **(D)** and epiphysis **(E)**. The growth plate should be differentiated from the joint space (*), which is located distal to the epiphysis. **b**. Longitudinal sonographic view of the knee joint in a 5 years old girl. Distal femoral epiphysis **(E)** is visualized distal to the diaphysis **(D)**. The epiphysis shows peripheral non-ossified cartilage **(c)**. The articular cartilage is seen as a superficial echogenic line overlying the cartilage (arrow). The differentiation of the joint components is enhanced by the acoustic window provided by a significant joint effusion **(F)**.

The growth plate is seen as a hypo-echoic area (same echogenicity as cartilage), with ill-defined margins and signs of vascularization, located between the diaphysis and epiphysis (Figure 1a). An inexperienced operator may erroneously think that this area is the joint space, a fracture or erosion [6].

As previously mentioned, the epiphysis can show different degrees of calcification, from an ossification center surrounded by cartilage (Figure 1b) to a completely ossified area. The articular cartilage can be seen occasionally (Figure 1b). Joint effusion may aloud better visualization of these structures (Figure 1b).

### Knee joint

**Figure 2:**
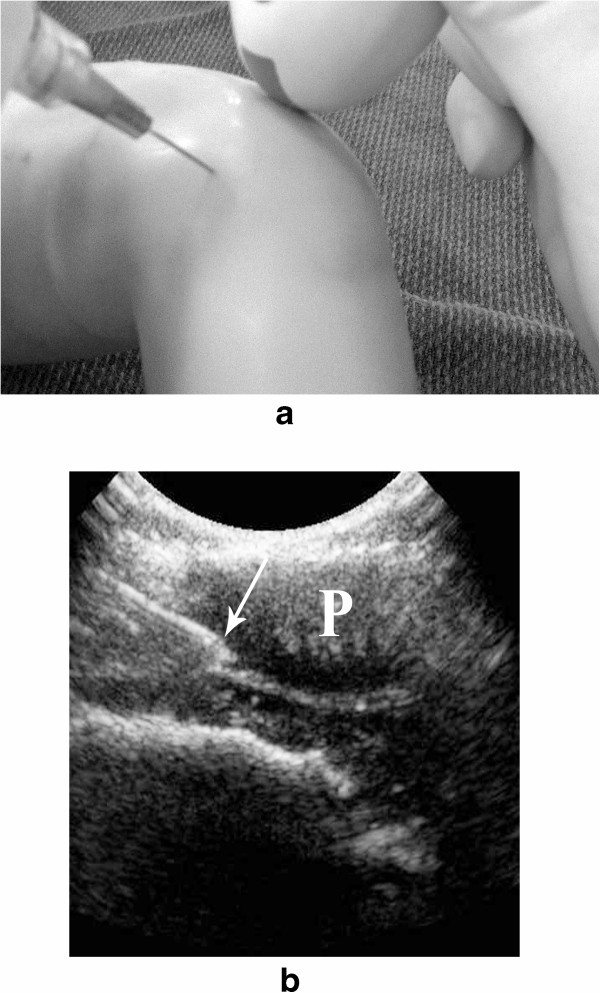
**Ultrasound guided steroid injection of the knee. a**. Simulated injection in a mannequin showing the advancement of the needle using an “in-plane” approach with the ultrasound probe. **b**. Injection in a patient in which the non-ossified patella provides an excellent acoustic window for advancement of the needle (arrow) into the joint space.

The patient is positioned supine. The 8 MHz curved array transducer is our first choice for this joint. We consider that the medial and lateral aspect of the knee, inferior to the patella, is safe for steroid injections, taking into consideration that the popliteal vessels are located posteriorly. The injection can be done using anatomical landmarks or US guidance. When using anatomical landmarks the medial or lateral margin of the patella is palpated and the needle is advanced under it, with a mild anterior-posterior angulation. If ultrasound is used, the needle is advanced using an “in-plane” approach with the ultrasound probe (Figure 2a). In patients in whom the patella is not fully ossified, it can provide an excellent acoustic window for advancing the needle into the joint space (Figure 2b). When a significant amount of joint effusion is present, we routinely remove as much fluid as possible prior to the steroid injection and a supra-patellar recess approach can be used [4].

### Ankle joint

**Figure 3:**
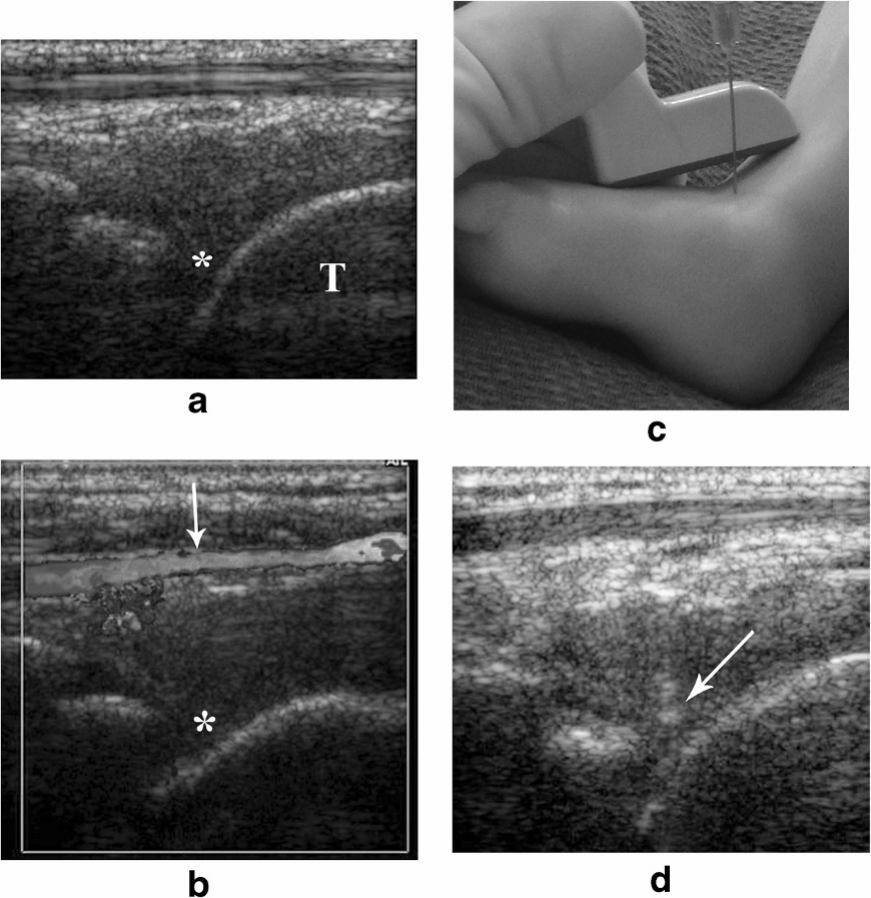
**Ultrasound guided steroid injection of the ankle. a**. The anterior portion of the ankle joint (*) is seen in between the distal tibial epiphysis and the talus (T). **b**. It is crucial to identify prior to the injection, the location of the anterior tibial artery becoming the dorsalis pedis artery (arrow), anterior to the ankle joint (*) using grey scale and color-Doppler. **c**. Simulated ankle joint injection in a mannequin showing the advancement of the needle using an “out-of-plane” approach. **d**. The needle tip is clearly visualized being advanced into the joint space (arrow).

The patient is positioned supine. The transducer of choice is the 15 MHz linear array (small foot print: “Hockey stick”). In teenagers a 12 MHz linear or 8 MHz curved array may be required. For this joint it is important to place the transducer in a longitudinal plane along the long axis to the tibia. It is then advanced inferiorly until the distal tibial growth plate and epiphysis are recognized (Figure 1a). Distal to this ephyfisis, the tibio-talar joint is seen. The anterior portion of this joint usually shows a “V” shape appearance (Figure 3a). This place is our ideal site for the injection. It is important to visualize the location of the anterior tibial artery becoming the dorsalis pedis artery anterior to the ankle joint (Figure 3b). Once this structure is recognized, the needle can be inserted in a spot medial or lateral to it. The transducer is placed along the long axis of the joint and the needle is advance perpendicular to it (“out-of-plane) (Figure 3c), until the tip is seen deep in the joint space (Figure 3d).

### Hip joint

**Figure 4:**
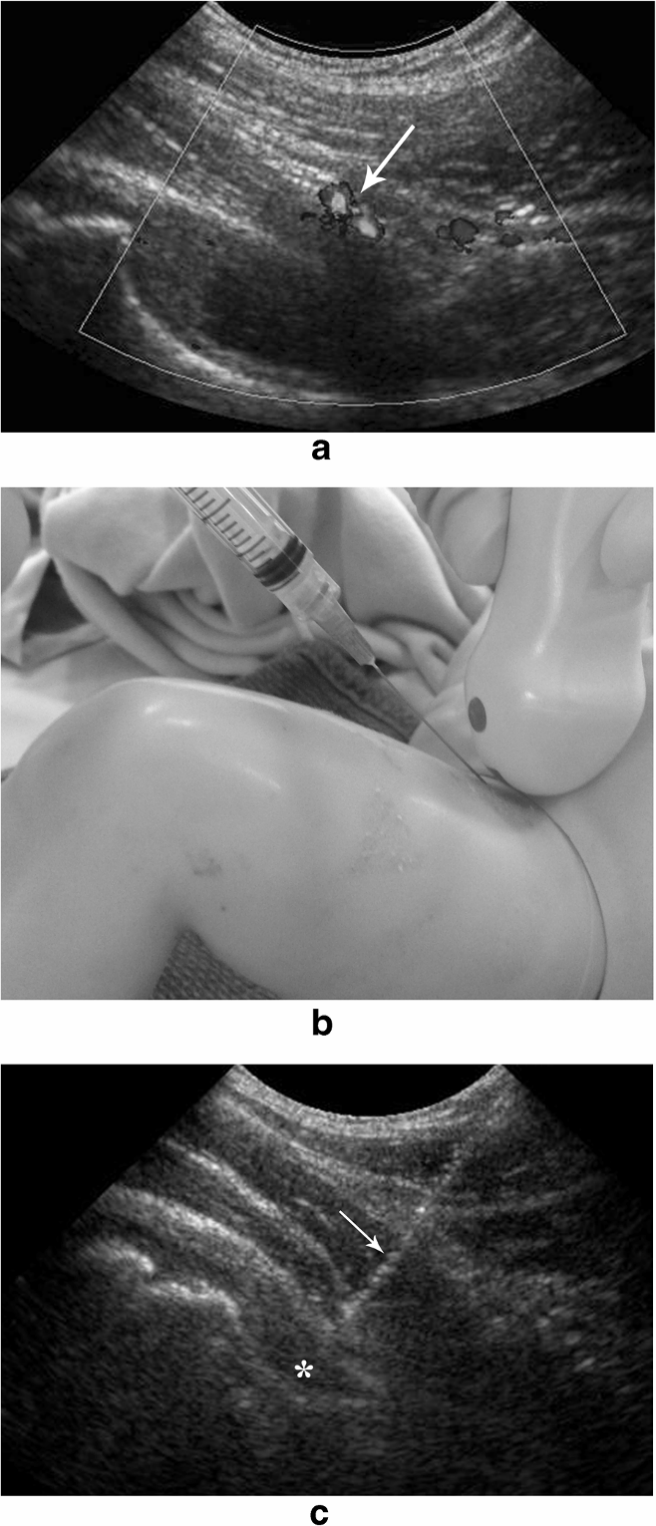
**Ultrasound guided steroid injections of the hip. a**. Longitudinal view of the hip joint and proximal femur which shows in the color-Doppler study the circumflex femoral vessels (arrow). **b**. Simulated hip joint injection in a mannequin showing the advancement of the needle using an “in plane” approach. **c**. Sonographic view of the needle (arrow) being advanced into the hip joint space (*).

The patient is positioned supine. The 8 MHz curved array transducer is our first choice for this joint. In teenagers and obese patients, a 5 MHz transducer may be required combined with a 25Gauge-3.5 inches spinal needle to reach the joint space (Spinal Needle, BD Medical, Franklin Lakes, NJ). Different degrees of ossification of the femoral head can be seen. The transducer is placed along the long axis of the femoral neck, a view that is frequently utilized in pediatric radiology for the diagnosis of joint effusion. We usually use a puncture site lateral to the femoral vessels. The color-Doppler study frequently shows the circumflex femoral vessels and its branches in the soft tissues anterior-inferior to the joint, which should be avoided (Figure 4a). The needle is directed to the lower aspect of the joint capsule; being advanced using an “in plane” approach (Figure 4b), keeping the femoral neck and joint space in the long axis (Figure 4c).

### Wrist joint

**Figure 5:**
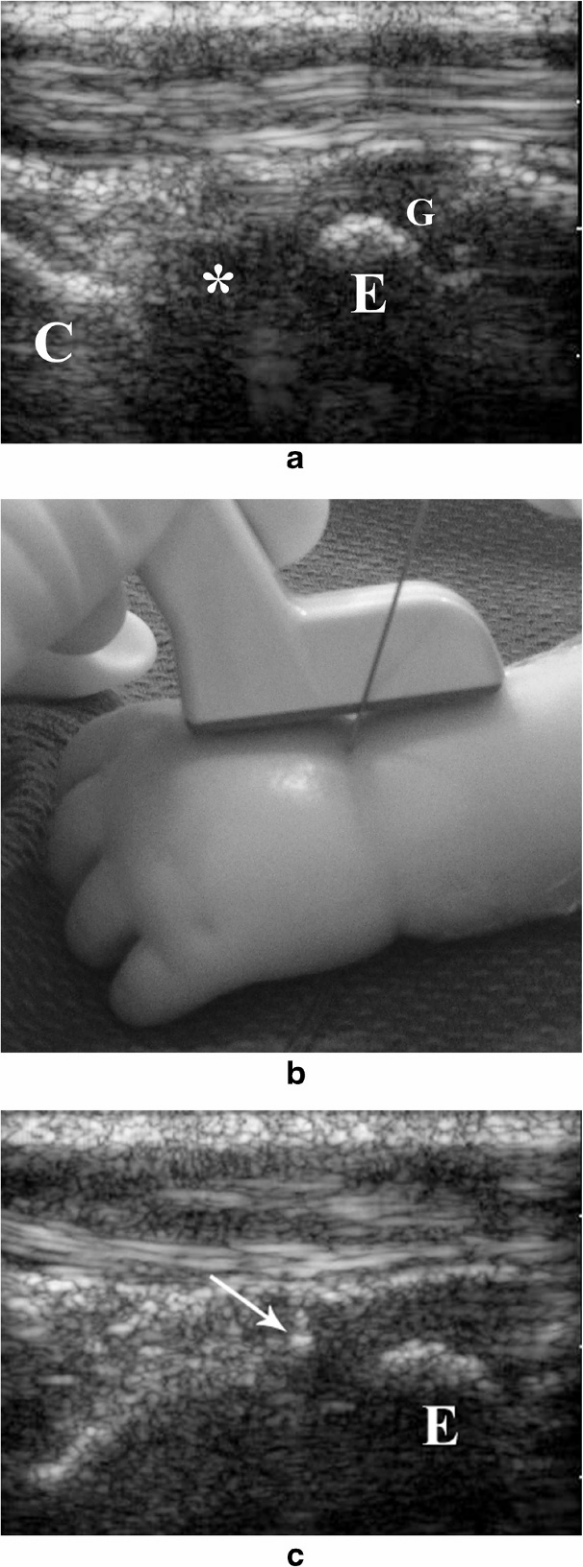
**Ultrasound guided steroid injection of the wrist joint. a**. The wrist joint (*) is seen placing the transducer in the longitudinal axis of the radius, in between the distal epiphysis of the radius (E) and the proximal carpal bones (C). It is important to differentiate the joint space (*) from the distal growth plate of the radius (G). **b**. Simulated wrist joint injection in a mannequin showing the advancement of the needle using an “out-of-plane” approach. **c.** The needle (arrow) is advanced into the joint space, keeping the distal radial epiphysis (E) as a reference.

We perform the injection though the dorsal aspect of the wrist, therefore the forearm is in a prone position and secured in place. The preferred transducer is the 15 MHz linear array. The transducer is placed in the long axis of the radius and then advanced distally until the distal growth plate and radio-carpal joint are identified (Figure 5a). The needle is advanced into the radio-carpal joint space having the distal radius as a reference. The transducer is placed along the long axis of the joint and the needle is advanced perpendicular to it (out-of-plane) (Figure 5b) until it reaches the joint space (Figure 5c).

### Inter-phalangeal joints

**Figure 6:**
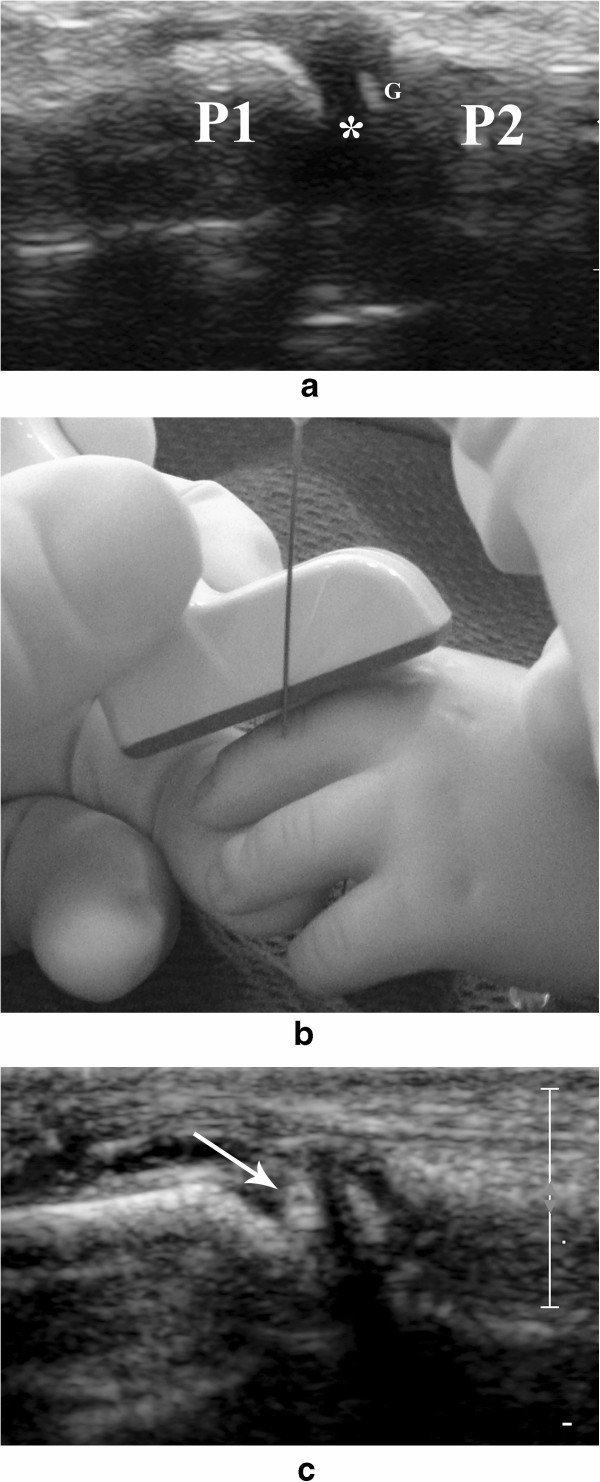
**Ultrasound guided steroid injection of the inter-phalangeal joints. a**. The joint space (*) is seen as a gap between the proximal (P1) and distal (P2) phalanx. It should be clearly differentiated from the growth plates (G) of these bones. **b**. Simulated inter-phalangeal joint injection in a mannequin showing the advancement of the needle using an “out-of-plane” approach. **c**. The needle (arrow) is seen being advanced into this small joint space.

We place the hand in the prone position. The transducer of choice is the 15 MHz linear array. The injection is performed in the dorsal aspect of the joint. The transducer is placed along the long axis of the finger and the joint is visualized as a gap between the two phalanges, being fundamental to clearly differentiate the growth plates from the joint space (Figure 6a). The needle is advanced perpendicular (out-of-plane) and very close to the transducer (Figure 6b), until its tip is visualized within the joint space (Figure 6c). Occasionally we inject metacarpo-phalangeal joints with US guidance and without the aid of fluoroscopy.

## Discussion

The developing bone and joints in children have a typical appearance which changes with the different stages of the growth. For the physicians performing joint injections in children it is very important to be familiar with these findings. The aim of this article was to describe the technique that we use to perform joint injections of a group of joints which most of the time can be injected using US as the only image guidance technique.

## Authors’ information

Pediatric Interventional radiologist at the Hospital for Sick Children, Toronto, Ontario, Canada. More than eight years of experience performing joint injections in a large and diverse population of patients with Juvenile Idiopathic Arthritis.

## Abbreviations

JIA: Juvenile idiopathic arthritis
US: Ultrasound
CT: Computed tomography.

## References

[1] Weiss J, Ilowite N (2005). Juvenile idiopathic arthritis. Pediatr Clin N Am.

[2] Manners PJ, Bowers C (2002). Worldwide prevalence of juvenile arthritis: why does it vary so much?. J Rheumatol.

[3] Hashkes P, Laxer R (2005). Medical treatment of Juvenile Idiopathic Arthritis. JAMA.

[4] Young C, Shields W, Coley B, Hogan MJ, Murakami JW, Jones K (2012). Ultrasound-guided corticosteroid injection theray for juvenile idiopathic arthritis: 12-year care experience. Pediatr Radiol.

[5] Bruyn G, Schmidt W (2009). How to perform ultrasound guided injections. Best Pract Res Clin Rheumatol.

[6] Karmazyn B (2011). Ultrasound of pediatric musculoskeletal disease: from head to toe. Semin Ultrasound CT MRI.

[7] Navarro O, Parra D (2009). Pediatric musculoskeletal ultrasound. Ultrasound Clin.

